# PSYCHOCOGNITIVE REACTIVITY TO INSUFFICIENT SLEEP AND ITS ASSOCIATION WITH BODY MASS INDEX IN MIDDLE-AGED WORKERS

**DOI:** 10.1093/geroni/igz038.2383

**Published:** 2019-11-08

**Authors:** Taylor Drury, Soomi Lee, Orfeu M Buxton, David M Almeida

**Affiliations:** 1 University of South Florida, Tampa, Florida, United States; 2 Pennsylvania State University, University Park, Pennsylvania, United States

## Abstract

Individuals tend to report more stressors on days after nights with fewer hours of sleep. There may be individual differences such that this negative sleep duration—stressor perception relationship is stronger for some than others, which may have implications for health outcomes. However, we know little about whether differences in stressor perception in response to insufficient sleep (“psychocognitive reactivity to insufficient sleep”) are associated with health outcomes such as body weight. This study examined whether psychocognitive reactivity to insufficient sleep were associated with body mass index (BMI) in midlife workers. We used a sample of 127 office workers (Mage=45.2±6.2) who participated in a daily diary study for 8 consecutive days as part of the Work, Family, and Health Study. Multilevel models tested whether daily number of stressors was predicted by previous nights’ sleep. We outputted within-person slopes of stressors regressed on sleep duration to predict BMI (kg/m2). Analyses adjusted for sociodemographic characteristics and mean stressors across days. On average, workers reported more stressors following nights with shorter sleep duration than usual (negative slope means higher reactivity). Compared to those with average reactivity (within ±½SD; reference), workers with higher reactivity (≤-½SD) had higher BMI (p<.05). The BMI of these workers fell in the obese range. This study is one of the first to report that middle-aged workers with higher psychocognitive reactivity to insufficient sleep may be at greater risk for obesity. Future interventions should focus on improving middle-aged workers’ sleep health to reduce next-day stressors and thereby improve their body weight.

